# Multimedia-assisted instruction on pain assessment learning of new nurses: a quasi-experimental study

**DOI:** 10.1186/s12909-019-1496-z

**Published:** 2019-03-04

**Authors:** Tsung-Lan Chu, Jeng Wang, Hui-Ling Lin, Hsiu-Fang Lee, Chiu-Tzu Lin, Li-Yu Chieh, Yu-Chih Sung, Yueh-E Lin

**Affiliations:** 1 Chung Gung Memorial Hospital, Department of Quality Management, Taoyuan, Taiwan; 2 Department of Nursing, Chang Gung University of Science & Technology, Taoyuan, Taiwan; 3 Department of Nursing, Linkou Chang Gung Memorial Hospital, No.5, Fuxing St., Guishan Dist, Taoyuan City, 333 Taiwan; 4 School of Nursing, Chang Gung University, Taoyuan, Taiwan; 5 School of Nursing, National Taipei University of Nursing and Health Sciences, Taipei, Taiwan; 6 Chang Gung Medical Education Research Centre (CG-MERC), Taoyuan, Taiwan

**Keywords:** Pain assessment, Multimedia, teaching materials, Nursing education

## Abstract

**Background:**

Pain assessment and treatment are key factors affecting the quality and safety of care for patients and capabilities related to them are crucial for new nursing staff. Consequently, we developed a multimedia-assisted teaching program for nursing newcomers’ pain assessment learning to facilitate their practical pain assessment ability. The goal of this study was to evaluate a multimedia instructional program to boost new nurses’ ability to conduct pain assessment and treatment, through simulated scenario instruction.

**Methods:**

A quasi-experimental, pretest-posttest design with purposive sampling was used in this study. Eighty-six nurses were enrolled (control group, *n* = 39; experimental group, *n* = 47). Both groups underwent traditional pain assessment training in the classroom. The control group received lectures using PowerPoint files; while the experimental group undertook pain assessment training with the same content but delivered via multimedia-assisted instruction based on the ADDIE model. Pre- and post-instruction questionnaires relating to pain knowledge were completed. Participants’ competence in performing pain assessment was subsequently evaluated one-month post instruction.

**Results:**

The experimental group had significantly higher satisfaction scores (27.67 ± 3.76 vs. 31.36 ± 3.42, *p* < .01, respectively), and demonstrated greater knowledge of pain assessment (7.73 ± 0.67 vs. 7.08 ± 0.90, *p* < .05, respectively) than did the control group. Additionally, when evaluated at the one month follow-up, newcomers in the experimental group had better communication ability to perform pain assessment (26.58 ± 3.01 vs. 25.08 ± 3.32, *p* < .05, respectively).

**Conclusions:**

The program can improve nurses’ pain assessment knowledge and competence. Newcomers were able to better respond to patients in pain, which is essential for pain assessment. This pilot study thus suggests a new, multimedia program for training nursing newcomers in pain assessment.

## Background

Pain is often considered the fifth vital sign. Acute pain accounts for over 80% of physician visits [1]. Pain assessment is complex because pain is a subjective experience, which cannot be measured by objective instruments. Therefore, pain assessment is the most difficult among vital signs assessments for new nurses in clinical care practice [2]. Further, pain assessment plays a key role in guiding therapy to provide pain relief, and proper pain relief is essential for patients [3]. Nurses are recognized as playing a key role in pain management because they are the first-line medical staff in providing patient care. Therefore, pain assessment and care are essential skills for nursing staff as knowledge of pain assessment and treatment is critical for patients’ quality of care [4]. However, inadequate knowledge of pain management is common among nurses [5]. Factors such as unfamiliarity with the workplace, insufficient professional knowledge, lack of relevant clinical experiences, and lack of professional confidence [6] indicate that new nurses in particular may be unable to provide the pain assessment and treatment that patients require.

Löfmark, Gustavsson, and Wikblad investigated student nurses’ ability to perform pain assessment, and found that two-thirds of student nurses did not show adequate knowledge and skill in pain assessment and handled their assessments casually [7]. However, the ability to correctly assess patients’ pain experience and provide correct treatment is essential, and insufficient pain assessment knowledge is a critical factor that leads to inadequate pain management [8]. It is well-known that there is a gap between theory and practice in this regard, and how to narrow or eliminate the gap is still a major task in nursing schools and nurses’ workplaces [7]. Aziato and Adejumo suggested that insufficient pain management knowledge may result from gaps in nurses’ education and recommended that pain assessment courses should be included in nursing curricula [9]. Consequently, many institutions arrange for pain education for new nurses to boost their pain-related professional skills, promote learning of pain assessment and treatment skills, and collect pain-related information, which helps nurses provide necessary treatment for pain at appropriate times [6].

Via proper pain education, nurses’ knowledge, management, attitude, and assessment techniques related to pain can be improved [10]. Further, pain education can reduce stressors, improve coping skills, and boost appraisal ability [11]. Education not only enhances the professional care knowledge of medical personnel, it also increases patient safety. Apart from ensuring that student nurses receive correct pain education, it is recommended that practicing nurses should be monitored and appraised regularly to ensure that they have fully absorbed pain management training [9]. According to the findings of Horbury et al., an innovative teaching method or training program is needed in the clinical education field to raise nursing staff’s knowledge of their deficiency in pain assessment and management [4]. The use of professional blended e-learning instruction can help learners adapt to the workplace and engage in lifelong learning [12]. A lively, multimedia interface can attract learners’ attention, thereby boosting learning efficiency, and adding interaction practice questions enables learners’ level of understanding to be evaluated [12].

Repeated practice can promote learners’ ability to memorize particular movements correctly; enhance long-term memory toward achieving training goals; and increase learners’ ability to think, judge, and problem solve [13]. In addition, repeated scenario stimulation learning not only boosts learners’ confidence, but also increases learners’ interest, which in turn encourages active learning and improves effectiveness [14]. A survey conducted by Abdalrahim et al. revealed that a teaching approach whereby senior nurses led junior nurses, using of one-way in-service instruction to help the newcomers understand pain assessment and treatment, did not sufficiently meet the needs of new nurses whereas blended instruction, applying multimedia and various scenario cases, was highly successful: enabling learners to engage at any time, and facilitating new nurses’ pain assessment learning and treatment skills [10]. Twycross recommended that efforts to improve pain treatment education should first assess existing pain management education strategies to gain a better understanding of nurses’ patient care knowledge and skills [15].

Multimedia instruction occurs in a networked environment and relies on computers and other equipment to transmit information. It employs a combination of media materials, demonstrations, and case instruction via an open-media system [16, 17]. Apart from their use in clinical care, e-learning and computer learning have become an indispensable capability for both learners and instructors [18]. Multimedia promotes learners’ cognition and learning motivation, but must avoid excessive stimulation and learning overload [19]. Multimedia instruction provides information effectively, improves learners’ understanding of and adherence to the instructional content by reducing learning anxiety, and can reduce associated costs [20]. Consequently, multimedia instruction is widely used in various types of health education, mainly to provide instruction on care before and after treatment [21].

Multimedia instruction uses various approaches to transform tedious or dry knowledge into more interesting forms, which can induce learners to actively participate in learning; it multimedia instruction changes passive learning into active learning, while broadening the scope of thinking and imagination [16, 17]. Baraz et al. noted that multimedia instruction improved individual knowledge and social behavior among patients with diabetes [22]. A study of multimedia learning modules found that 87.1% of learners receiving multimedia instruction believed that visual assistance helped them obtain a more realistic learning experience; 77.4% of learners agreed that multimedia deepens understanding of the topics to be learned, and that video or animated graphics provide even more detailed information and facilitate faster learning; and 80.6% of learners agreed that the use of animation and recorded audio increases memory retention [23]. A multimedia instruction strategy has been demonstrated to boost learning motivation, enhance nurses’ professional skills and problem-solving ability, and impart the professional capabilities needed for work [24]. Interactive instruction that incorporates multimedia may also enhance learners’ attitudes toward learning, which will also influence learning effectiveness [25, 26]. At the same time, conducting an instruction via multimedia increases learning satisfaction and self-efficacy [27], and is effective in reducing patients’ pain, uncertainty, and anxiety [21]. Consequently, the goal of this study was to design a multimedia instructional approach suitable for teaching new nurses pain knowledge and skills and to boost nurses’ ability to conduct pain assessment and treatment, through scenario simulation instruction.

## Methods

### Education intervention design

The design and development of the multimedia pain assessment instruction video was based on the five components of the ADDIE instructional model—Analysis, Design, Development, Implementation, and Evaluation [28].

At the “Analysis” step, we summarized the previous year pain assessment training records relating to the new nurses’ ability to handle pain assessment. The purpose of this step was to identify learner’s existing knowledge, skills, and obstacles of performing pain assessment, and previous pain assessment experience. We also identified the newcomers’ needs for pain assessment skills, and recommendations for performing pain assessment training courses. Second, the “Design” phase included the following content for instruction: importance of pain assessment and treatment, understanding and assessing pain, probable causes of pain, and treatment methods. Following this, during the “Development” phase, we invited academic experts with pain research experience, practical specialists, and senior clinical instructors to provide feedback on the instruction materials, and revised the instruction plan in accordance with this feedback prior to the “Implementation” phase. The content validity index (CVI) of the experts’ opinions was 0.95, and the instructional content was scanned as online pain instructional scenario simulation multimedia materials and interactive instructional content. The last phase, “Evaluation,” included evaluating learner’s satisfaction, knowledge, and ability to conduct pain assessment after using multimedia instruction.

### Participants and recruitment

Prior to approaching participants, ethical approval was obtained from the Change Gung Medical Foundation Institutional Reviewed Committee Board(No. 104-7209B). Participation inclusion criteria were (1) new nursing staff (within 3 Months), (2) aged ≥20 years, and (3) willing to sign a consent form to participate in the study. One hundred and fifty one new nursing staff in the hospital were invited in this study. All new nursing staff were eligible to participate. Convenience sampling was employed. No pain instruction was given prior to consent being gained. Participating nurses consented to join the pain assessment training course, evaluate the course’s learning effectiveness, and be appraised for their ability to conduct pain assessment by their unit head nurse. Acceptance of control group members, who received ordinary classroom pain instruction with lectures using PowerPoint (PPT), was performed first in order to avoid participant cross contamination. Their data were collected from May 5 to May 30, 2016. Data for the experimental group, who received ordinary classroom instruction with multimedia simulation scenario pain teaching materials, was collected from November 1 to December 5, 2016. The nurses participating in the course could decide individually whether to participate in the study and complete the questionnaires. All the participants recruited in this study received study compensation (i.e., NTD100). The participating personnel did not know whether they belonged to the control group or the experimental group. All data were numerically encoded to ensure confidentiality.

Eighty-six junior nurses participated in the study (aged 23–25 years). The proportions of control group (*n* = 39) and experimental group (*n* = 47) were 45 and 55% respectively. They mainly came from the units of internal medicine, surgery, and gynecology/pediatric specialties. Most participants had experiences dealing with pain treatment but had not obtained pain assessment-related training. No significant differences were found between the groups’ basic characteristics (Table 1).

**Table 1:** New Nurses’ Demographic and Work-related Information

Variable	Control group (n = 39) Mean ± SD	Experimental group (n = 47) Mean ± SD	*t*/x^2^	*P* value (2-tailed)
Age (years)	23.21 (2.26)	23.03 (2.06)	.397	.692
Education level			.013	1.000
Junior college	7	8		
University	32	39		
Work attributes			7.127	.129
Internal medicine	9	11		
Surgery	15	7		
Gynecology/pediatrics	9	15		
Operating room	3	7		
Another unit	3	7		
Received in-service pain education course			3.162	.114
Yes	18	13		
No	21	34		
Pain treatment experience			.701	.623
Yes	38	44		
No	1	3		

*SD* standard deviation

### Study design

**Fig. 1:**
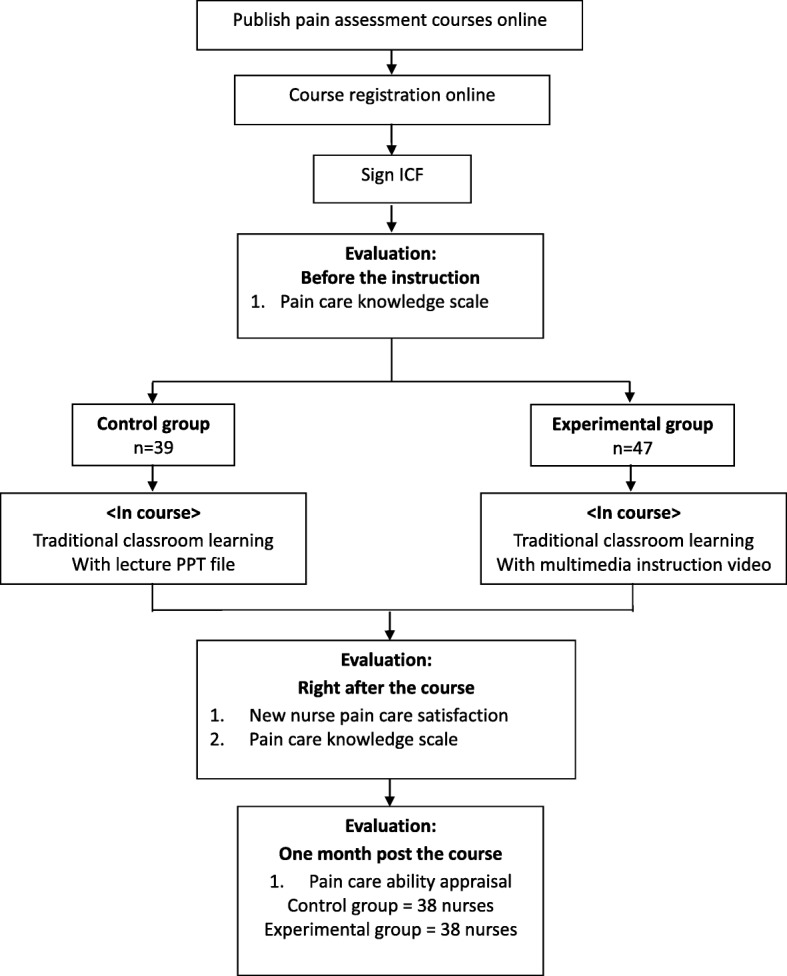
Research procedure

We employed a quasi-experimental, single blind, pre-post design. We also explored the effects of the multimedia pain instruction intervention on nurses’ clinical pain assessment and pain care ability one-month post training. The members of both control and experimental groups comprised new nursing personnel receiving approximately 35 min (70%) classroom instruction in pain assessment and care, followed by a 15 min (30%) case study section. The case study section in the control group was performed via lectures PPT solely by description of brief patient conditions and recommendation for pain assessment. While in the experimental group, the case study section was performed via multimedia with pain scenarios followed by interactive discussion on pain assessment. Both groups completed the new nurse pain care learning satisfaction scale and the pain care knowledge scale after instruction. One-month post training, the participating nurses’ unit head nurses assessed the learning effectiveness of the program, by verifying the learners’ pain care ability (Fig. 1).

### Instruments

We utilized three instruments to examine study variables.

New nurse pain care satisfaction. This section included 7 items concerning participants’ self-assessed satisfaction with the course after receiving pain instruction. The items were scored using a 5-point Likert scale (1–5); higher scores indicated better self-assessed satisfaction with pain instruction. Two nursing faculty members with Ph.D. degrees and three clinical specialists were invited to inspect this section, which obtained a CVI value of 0.92 and a Cronbach’s α of 0.96.

Pain care knowledge scale. The pain care knowledge scale was based on a scale developed by Wu et al. [29] that sought to measure nurses’ pain care knowledge and techniques. This scale contains 3 subscales addressing pain assessment, pain treatment, and drug treatment. It comprises 9 items, with each subscale consisting of 3 questions. Correct answers scored 1 point, and incorrect answers scored 0. This scale had a CVI value of 0.95 and an overall Cronbach’s α of 0.77. Two nursing faculty members with Ph.D. degrees and three clinical specialists assessed the scale, which had a CVI value of 0.95 and a Cronbach’s α of 0.85. The participants completed the items for this scale before and again after instruction.

Pain care ability appraisal. A Mini Clinical Evaluation Exercise for Trainees (Mini-CEX) pain care scale, developed based on nursing instruction as conducted at Chang Gung Memorial Hospital, was used to appraise pain care ability. It contained two parts, respectively concerning professional pain care capabilities and professional communication ability. The questions were scored on a 5-point scale, where a higher score indicated a more positive result. Two nursing faculty members with Ph.D. degrees and three clinical specialists assessed the scale, which had a CVI value of 0.92 and a Cronbach’s α of 0.87. This appraisal was conducted by the participating nurses’ head nurse to evaluate their pain care ability one month after the instruction.

### Statistical analyses

The Student’s t-test was used to analyze basic attributes, while t-tests and paired t-tests were used to analyze differences between the two groups. Alpha was set at *p* < .05.

## Results

### New nurses’ satisfaction with pain instruction

The satisfaction scores in both groups reflected the overall training instruction experience. The experimental group perceived higher level of satisfaction for assessment training than did the control group (Table 2). The experimental group thus viewed themselves as more able to correctly assess pain symptoms and signs, use appropriate nursing measures in the pain care process, and actively help patients deal with pain.

**Table 2:** New Nurses’ Satisfaction with the Pain Instruction

Question	Control group (n = 39) Mean ± SD	Experimental group (n = 47) Mean ± SD	*t*-value
1. Able to correctly assess pain symptoms and signs	4.00 ± 0.56	4.47 ± 0.55	3.90**
2. Able to select appropriate nursing guidelines in the pain care process	3.77 ± 0.74	4.51 ± 0.51	5.49**
3. Able to select appropriate nursing measures in the pain care process	3.85 ± 0.67	4.47 ± 0.50	4.91**
4. Able to give directions effectively for medication use methods in the pain treatment process	4.03 ± 0.54	4.43 ± 0.50	3.55**
5. Can use non-drug methods effectively in the pain treatment process	3.87 ± 0.70	4.49 ± 0.55	4.61**
6. Able to track patients’ responses following treatment for pain	4.08 ± 0.62	4.51 ± 0.51	3.56**
7. Able to actively help patients deal with pain.	4.08 ± 0.77	4.49 ± 0.51	2.97*
Overall satisfaction	27.67 ± 3.76	31.36 ± 3.42	4.78**

*SD* standard deviation; *p < .05, **p < .01

Changes in Knowledge Between the Two Groups After the Program.

Prior to pain instruction, there was no significant difference between the two groups regarding mean pain knowledge score (Table 3); however, after the intervention, the two groups differed significantly on the topic of pain assessment and overall score, but showed no difference in the other two topics, pain treatment and pain medicine.

**Table 3:** Knowledge Scale Pre-test scores of Both Groups Prior to Receiving Pain Instruction

Topic	No. of questions	Control group (n = 39) Mean ± SD	Experimental group (n = 47) Mean ± SD	t-value
1. Pain assessment	3	2.41 ± 0.50	2.61 ± 0.49	1.80
2. Pain treatment	3	1.72 ± 0.72	1.81 ± 0.74	0.53
3. Pain medication	3	2.77 ± 0.43	2.67 ± 0.52	1.03
Overall scale		6.90 ± 0.97	7.07 ± 0.93	0.83

*SD* standard deviation, *No.* number

Both the control and experiment group knowledge scores differed over time, in other words, between pre- and post- scores. The knowledge scores for both groups increased; however, the experimental group had significantly better post-test scores than did the control group (Table 4).

**Table 4:** The Pain Knowledge Scores of Both Groups Before and After Receiving Pain Instruction

Topic	Control group (n = 39)				Experimental group (n = 47)			
	Pre-test Mean ± SD	Post-test Mean ± SD	Improve Mean ± SD	t-value	Pre-test Mean ± SD	Post-test Mean ± SD	Improve Mean ± SD	t-value
1. Pain assessment	2.41 ± 0.50	2.44 ± 0.64	0.03 ± 0.58	0.27	2.61 ± 0.49	2.80 ± 0.40	0.20 ± 0.51	2.45*
2. Pain treatment	1.72 ± 0.72	1.85 ± 0.67	0.13 ± 0.83	0.96	1.81 ± 0.74	2.00 ± 0.51	0.19 ± 0.74	1.77
3. Pain medication	2.77 ± 0.43	2.79 ± 0.47	0.03 ± 0.58	0.27	2.67 ± 0.52	2.83 ± 0.38	0.15 ± 0.56	1.86
Overall scale	6.90 ± 0.97	7.08 ± 0.90	0.18 ± 1.12	1.00	7.07 ± 0.93	7.73 ± 0.67	0.66 ± 0.88	4.77**

*Improve* improvement between tests, *SD* standard deviation; *p < .05, **p < .01

### Follow-up appraisal of pain care ability

One month after the new nurses received pain education, a follow-up appraisal by the head nurses of their units was undertaken. This comprised an investigation of professional pain capabilities and communication ability and involved 38 nurses in each of the control and experimental groups (some learners were not included because they were operating room, nursing home, or pediatric intensive care nurses and thus did not perform pain assessments). There was no significant difference found between the two groups in professional capabilities; however, the experimental group presented significantly higher scores for communication ability than the controls did. Of note is that a significant difference was also found between groups on the overall scale score (Table 5).

**Table 5:** The Appraisals of New Nurses’ Pain Care Ability by Unit Head Nurse After Receiving Pain Instruction

Topic	Control group (n = 38) Mean ± SD	Experimental group (n = 38) Mean ± SD	t-value
A. Professional capabilities			
1. Performing pain assessment using language and assessment instruments understood by patients	4.34 ± 0.53	4.45 ± 0.65	.78
2. Interacting with patients, able to listen patiently, having empathy	4.32 ± 0.57	4.53 ± 0.60	1.56
3. Able to assess patients’ pain histories, pain characteristics, and physiological influence	4.11 ± 0.65	4.24 ± 0.63	.89
B. Communication ability			
1. Able to convey a respectful and considerate attitude when interacting with patients	4.13 ± 0.74	4.50 ± 0.56	2.45*
2. Able to actively and promptly respond to patients’ pain needs and feelings	4.11 ± 0.73	4.42 ± 0.60	2.07*
3. Uses language understood by patients to explain pain measures	4.08 ± 0.63	4.75 ± 0.60	2.60*
Overall scale	25.08 ± 3.32	26.58 ± 3.01	2.06*

*p < .05, **p < .01

## Discussion

The present study employed classroom instruction on pain care supplemented with multimedia instructional DVDs as interventional teaching materials. The pain assessment and treatment instructional system developed in this study included pain concepts, assessment, and handling units, and incorporated case studies and discussion. In addition, text, images, instructional videos, and audio recordings were employed as multimedia instructional tools. Most of the new nurses in the experimental group had a positive impression of the content of the multimedia instructional DVDs and found that the multimedia instructional approach facilitated concentration and learning satisfaction. Furthermore, it was found that they could better perform correct assessment, implement care measures, and track the reactions of patients receiving treatment for pain, and that they felt greater satisfaction with their ability to help patients deal with pain than the control group. This finding was consistent with the conclusion of Zhang [27]—that interactive multimedia instruction provides learners with high satisfaction with the learning process and high self-efficacy, and the suggestions of Leow and Neo and of others that the images, animation, and audio of multimedia instruction helps learners to obtain a better learning experience, and deepens knowledge of the topics learned [12, 23]. The results of this study that classroom teaching paired with auxiliary DVD instruction is an effective way of enhancing nurses’ learning. This is consistent with the proposal of Liaw et al. that multimedia instruction can stimulate learning motivation, deepen acquisition of knowledge and skills, and thereby promote learning of work-related skills [24].

However, while the experimental group had a significantly higher pain assessment post-test score than the control group, no significant difference was found between the experimental group and control group for the aspects of pain treatment and pain medication. This result may be because the content of pain treatment and pain medication were both covered in the 35-min classroom instruction in pain assessment and care, instead of in the case study section which differed the intervention of both group in this study. Alternatively, or also, the new nursing staff may have been especially focused on how to conduct pain assessment, but concomitantly neglected pain treatment and medication-related knowledge. Before caring for patients with pain, apart from gaining a familiarity with pain care tasks, nurses should also understand relevant drug mechanisms and principles. Consequently, instruction in the pharmacology of pain medications must also be provided to new nurses to strengthen their understanding of and ability to apply pain treatments and medications.

In the present study, unit head nurses were asked to appraise new nurses’ care ability following ordinary classroom instruction with multimedia pain education. New nurses receiving multimedia instruction displayed significantly better pain care skills and ability to give explanations and communicate with patients, which indicates that receiving multimedia instruction enabled the nurses to understand pain care based on scenarios simulated from case studies. In addition, new nurses may need some time to internalize professional knowledge and develop sufficient understanding how to deal with patients’ pain problems. The absorption of professional skills requires constant exercise of those skills, and cross-sectional appraisals or appraisals performed at one month after the end of instruction may not necessarily reveal actual longer-term learning effectiveness. Finally, the length of the intervention course might limit learning [30].

To gain a better understanding of the effectiveness of instruction, patients may possibly be enlisted to supplement or assist with learners’ appraisal. However, the fact that the experimental group displayed better professional communication abilities and attitude than the control group shows that a multimedia pain education program can enhance learners’ knowledge, attitude, and ability to put knowledge to practical use [10, 26]. Muthulakshmi and Veliappan similarly suggested that multimedia instruction enhances learners’ learning attitude and thereby boosts their professional knowledge [25]. Likewise, Romero-Hall proposed that e-learning can enhance pain education learning and behavior [8]. We suggest that a longitudinal study a including patients to assist with the appraisal of learners’ pain care ability will yield more objective results.

In summary, the simultaneous implementation of multimedia instruction and classroom teaching can indeed boost nurses’ levels of knowledge more effective than conventional alternatives. The use of multimedia-assisted instruction accords with nurses’ learning styles and actual work needs, and enhances nurses’ learning concentration, satisfaction, pain-related knowledge, and pain care ability.

### Limitations and strengths

The limitations of this study include the fact that participants were all from a single medical center, which limits the extensibility of the results to all new nurses receiving multimedia pain instruction. Also, convenience sampling was performed among all new nurses receiving in-service pain education, which may bias the data. In addition, the one month follow up rate for experimental group was lower (*N* = 47 to 38, 81%) than the control group (*N* = 39 to 38, 97%). There might be an inherent selection bias in running the control group in the spring and the experimental group in the winter. Nevertheless, the results further our understanding of the effect of multimedia instruction on learning effectiveness, and therefore constitute a key reference for improving the quality of pain instruction. We recommend that future studies recruit participants from multiple hospitals. In addition, the cross-sectional framework used here may not shed light on participants’ subsequent ability to apply what they have learned; therefore, we recommend that future studies apply a longitudinal research design and enlist the assistance of patients in appraising nurses’ learning effectiveness. Together, these measures may improve understanding of learning and utilization and provide a more comprehensive instructional reference.

## Conclusions

The multimedia pain instruction course employed as an intervention in this study was able to enhance new nurses’ learning satisfaction, bring about a significant improvement in pain assessment-related knowledge and facilitate new nurses’ ability to communicate with patients when providing pain care. Most of the new nurses in the experimental group assessed the multimedia instruction positively and felt that the instructional DVD content was comprehensive, appealing, and facilitated focus on learning. The more interactive scenario-based DVD instruction is therefore recommended to replace the lecture-based PPT instruction.

Recommendations concerning procedures and results are as follows. First, in view of the complexity of clinical pain situations, to better meet nurses’ pain assessment learning needs, apart from instruction on the theoretical aspects of pain, diverse instructional strategies can be employed, and may incorporate diverse clinical pain scenarios and examination questions along with a question-and-answer design. Second, since the participants were new nurses at a single medical center, we recommend that future studies expand their research scope and investigate differences in the pain care ability of nurses in different units, geographical regions, and levels of hospital. Third, while the multimedia instruction used in this study employed fixed clinical scenarios, allowing learners to repeatedly practice in certain situations, the situations seen in clinical practice are exceptionally diverse, and nurses’ ability to interact and communicate with patients when performing pain care may not necessarily be adequately improved through only a single course. We recommend that long-term tracking and multi-aspect appraisal be performed in future studies when investigating the effectiveness of instruction to boost nurses’ professional pain care qualifications. Last, since the nurses had a positive impression of the multimedia DVDs, we recommend that this instructional approach be extended to courses evaluating diverse diseases, to enhance nurses’ learning willingness and acquisition of skills in those contexts as well.

## Abbreviations

ADDIE: A instructional model theory which includes the processes of Analysis, Design, Development, Implementation, and Evaluation
CVI: Content validity index
DVD: Digital versatile disk
